# Children's hospitalizations with a mood disorder diagnosis in general hospitals in the united states 2000-2006

**DOI:** 10.1186/1753-2000-5-27

**Published:** 2011-08-07

**Authors:** Tamar Lasky, Aliza Krieger, Anne Elixhauser, Benedetto Vitiello

**Affiliations:** 1 MIE Resources, Kingston, Rhode Island, USA; 2 Zambarano Unit, Eleanor Slater Hospital, Cranston, Rhode Island, USA; 3 Center for Delivery, Organization, and Markets, Agency for Healthcare Research and Quality, Rockville, MD, USA; 4 Child & Adolescent Treatment & Preventive Intervention Research Branch, National Institute of Mental Health, Bethesda, MD, USA

## Abstract

**Background:**

Mood disorders including depression and bipolar disorders are a major cause of morbidity in childhood and adolescence, and hospitalizations for mood disorders are the leading diagnosis for all hospitalizations in general hospitals for children age 13 to 17. We describe characteristics of these hospitalizations in the U.S. focusing on duration of stay, charges, and geographic variation.

**Methods:**

The Kids' Inpatient Database was analyzed to calculate hospitalization rates for 2000, 2003, and 2006. For each year, information was available for over 2 million hospitalizations, representing 6.3 to 6.5 million hospital stays annually in acute care, non-psychiatric hospitals.

**Results:**

The rate of pediatric hospitalizations with a principal diagnosis of a mood disorder was 12.4/10,000 in 2000, 13.0 in 2003, and 12.1 in 2006. In the same period, the incidence of hospitalizations for depressive disorders decreased from 9.1 to 6.4/10,000 children while the incidence of hospitalizations for bipolar disorders increased from 3.3 to 5.7/10,000 children. The mean length of stay increased from 7.1 to 7.7 days, while inflation-adjusted hospital charges increased from $10,600 in 2000, to $13,700 in 2003, to $16,300 in 2006. The proportion of mood disorder stays paid by government increased from 35.3% to 45.2%. The Western region experienced the lowest rates (9.9/10,000, 11.6 and 10.2 in 2000, 2003 and 2006) while the Midwest had the highest rates (26.4, 27.6, and 25.4).

**Conclusions:**

Mood disorders are a major reason for hospitalization during development, especially in adolescence. Mood disorder hospitalizations remained relatively constant from 2000-2006, but diagnoses of depressive disorders decreased while diagnoses of bipolar disorders increased. Hospitalization rates vary widely by region of the country.

## Background

The impact of mood disorders in children has been described with respect to morbidity and mortality, with reports that, by age 18, 14.3% of adolescents will have experienced a mood disorder, that depression affects 1-2% of children 6-12 years old and 4-6% of adolescents 13-17 years old over a 12-month period, that depression is a primary risk factor for suicide, which is the third leading cause of death in adolescence, and that bipolar disorders have been increasingly diagnosed among children and adolescents [1-5]. While mood disorders in children are widely recognized to be associated with utilization of a full range of outpatient mental health services, it is less widely recognized that mood disorders are one of the leading diagnoses associated with children's admissions to general hospitals. In the United States, mood disorders were the second most frequent primary discharge diagnoses at age 10-14, and ranked first at age 15-17 out of all children's hospitalizations in general hospitals in 2000 [6,7]. We here report on the most recent trends in the rate of mood disorder hospitalizations in general non-psychiatric hospitals in the U.S. with the purpose of further documenting the relevance of these common disorders to child health.

Efforts to describe the burden of mental health conditions in children in the United States and the resources used to address this burden must rely on a variety of data sources reflecting the breadth of mental health services used to care for children with mental health problems [8]. Mental health services are provided in specialty mental health facilities, the general medical/primary care sector, the human services sector including schools and criminal justices systems, and through voluntary support networks [8]. Within the de facto mental health system, care is divided into public and private sectors with the public sector including federal and state resources, and the private sector including services operated by private agencies or financed with private resources. In 2003, public sources financed more than half of all spending for mental health in the U.S, with costs for inpatient services accounting for about one fourth of total mental health expenditure [9]. Hospitalization takes place in both specialty mental health facilities and general hospitals and covers a range of situations, from short term emergency management to long term institutionalization. Most hospitalizations for mental health occur in the non-specialty general hospitals in the U.S. [9].

Within this complex array of services, admissions to general hospitals are documented in a government run national probability-based sample of hospital stays through the Healthcare Cost and Utilization Project (HCUP) Kids' Inpatient Database (KID) that is released every three years. Researchers have used hospital discharge databases to describe children's hospitalizations for any psychiatric or mental health diagnoses, for intentional self-inflicted injuries, and for diagnoses of autism and attention-deficit hyperactivity disorder in the US [10-13]. Our analysis focuses on mood disorders because they are the largest category within hospitalizations with a mental health diagnosis in the database, and are the leading diagnosis associated with hospitalizations for children 15-17 of any diagnosis. By definition, the analyses presented here exclude hospitalizations with primary diagnoses of other mental health conditions such as: anxiety, somatoform, dissociative and personality disorders, schizophrenia, psychosis or substance related mental disorders.

The following questions were addressed: What was the rate of hospitalizations for children with a diagnosis of mood disorder over this period? How did the incidence of hospitalizations with depressive disorders vs. bipolar disorders change during this period? What were the patient and hospital characteristics of these hospitalizations with regards to age, gender, payer, charges and length of stay? What proportion of hospitalizations for mood disorders was associated with self-injurious/suicidal behavior? How did the incidence of children's hospitalizations for mood disorders vary in regions across the U.S.?

## Methods

The Kids' Inpatient Database (KID) is one in a family of databases and software tools developed as part of the Healthcare Cost and Utilization Project (HCUP), a Federal-State-Industry partnership sponsored by the Agency for Healthcare Research and Quality. The KID is a probability-based sample of pediatric stays from all hospitals that contribute data to HCUP. For each hospital, 10 percent of normal newborns and 80 percent of all other neonatal and pediatric stays are randomly selected. Weights are provided to allow the calculation of national estimates of hospitalizations in short-term, acute care hospitals (termed "community hospitals" by the American Hospital Association). Stays in specialized substance abuse and psychiatric facilities are excluded, but stays in psychiatric units within general hospitals are included. Information provided in the KID includes principal and secondary diagnoses, principal and secondary procedures, admission and discharge status, patient demographics (e.g., gender, age, race), total charges and length of stay. The KID is released every three years, and we used the years 2000, 2003, and 2006, the most recently available at the time [14]. The unit of analysis is a hospitalization, and it is possible that an individual patient contributes more than one hospitalization to the database in any given year. Hospitalizations are not linked by patient identifiers, and there is no way to analyze re-hospitalizations in this database.

HCUP uses the Clinical Classifications Software (CCS) tool for clustering patient diagnoses and procedures into a manageable number of clinically meaningful categories [15]. The Mental Health Substance Abuse Clinical Classification Software (CCS-MHSA) tool was integrated into the CCS in 2008, and we applied the CCS-MHSA software to the KID for 2000, 2003, and 2006 to report hospitalizations in their current classifications. We calculated national rates using weighted estimates derived from HCUP database for numerator data, and information from the US Census 2000, and population estimates for 2003 and 2006 for the denominators. The database offers the option of assessing hospitalizations by principal diagnosis or by any diagnosis, and each serves different purposes. The principal diagnosis is the condition which is the chief reason for the hospital stay, as determined after evaluation during the stay. To assess the overall burden of mood disorders we considered whether a child had any diagnosis of mood disorders. The CCS coding system assigns E codes (external cause of injury codes) to category 662, with the label "Suicide and Self-Inflicted Injury". The HCUP KID provides data on charges, the amount that hospitals billed for services. A ratio enabling calculation of costs is available for the 2003 and 2006 KID, but not the 2000 KID; to compare data over the study years we used charge data. To compare proportions of hospitalizations with different mental health diagnoses we used only the principal diagnosis because children may have more than one mental health diagnosis. We did not calculate incidence by race or ethnic groups because of the well documented concerns about states that do not report race or ethnicity [16]. Following technical recommendations provided by AHRQ's HCUP resources, the SAS 9.2 procedure, SURVEYMEANS, was used to calculate weighted estimates, accounting for the HCUP KID sampling methodology and using Taylor series estimation for the confidence intervals [17-19].

## Results

For each of the study years, information was available for over 2 million hospitalizations (unweighted) representing 6.3-6.5 million hospitalizations for children in the U.S., with fewer than 0.01% of cases missing information on diagnoses. In 2000, 2003 and 2006, the weighted number of hospitalizations of children under age 18 with a mental health principal diagnosis ranged from 145,024-160,252. The percentages of hospitalizations with a mental health principal diagnosis were 15.6%, 15.2%, and 15.0% in children 10-14 in the study years 2000, 2003, and 2006, and 15.2$, 14.5% and 13.7% in children 15-17 in the same study years. For children age 5-9, hospitalizations with a mental health principal diagnosis accounted for 4.8%, 4.4% and 4.7% of pediatric hospitalizations in the three study years. For children age 1-4, the percentages were 0.2% for each year.

Of the hospitalizations with a mental health principal diagnosis, 88,276 (55%) in 2000, 92,349 (60%) in 2003, and 86,251 (59%) in 2006 had a principal diagnosis of mood disorders. The incidence of hospitalizations with mood disorders as the principal diagnosis (MHSA-CCS code 657) was 12.4/10,000 (95%CI = 12.1-12.7) in 2000, 13.0/10,000 in 2003 (95% CI = 12.8-13.3), and 12.1/10,000 (95% CI = 11.9-12.2) in 2006. The incidence of hospitalizations with any diagnosis of mood disorders was 18.9/10,000 (95%. CI = 18.5-19.2) in 2000, 20.4/10,000 in 2003 (95% CI = 20.1-20.6), and 19.6/10,000 (95% CI = 19.3-19.9) in 2006.

The CCS-MHSA system subdivides the group "Mood disorders" into two categories, "Bipolar disorders" and "Depressive disorders." At this level of classification, the incidence of hospitalizations for depressive disorders decreased from 9.1/10,000 (95% CI = 8.8-9.3) in 2000, to 8.4/10,000 (95% CI = 8.3-8.6) in 2003, and to 6.4/10,000 (95% CI = 5.5-5.8) in 2006, while the incidence of hospitalizations for bipolar disorders increased from 3.3/10,000 (95%CI = 3.2-3.5) in 2000 to 4.6/10,000 (95% CI = 4.5-4.7) in 2003 and 5.7/10,000 (95% CI = 5.5-5.8) in 2006 (Table 1).

**Table 1:** Incidence of hospitalization per 10,000 and 95% Confidence Intervals among children under 18, 2000-2006

Diagnostic Category	2000	2003	2006
Mood disorders as principal diagnosis	12.4 (12.1-12.7)	13.0 (12.8-13.3)	12.1 (11.9-12.2)

Mood disorders as all-listed diagnosis	18.9 (18.5-19.2)	20.4 (20.1-20.6)	19.6 (19.3-19.9)

Bipolar disorders as principal diagnosis	3.3 (3.2-3.5)	4.6 (4.4-4.7)	5.7 (5.5-5.8)

Depressive disorders as principal diagnosis	9.1 (8.8-9.3)	8.4 (8.3-8.6)	6.4 (5.5-5.8)

At the most granular level, the category, "Mood disorders", includes 56 ICD-9-CM codes (Appendix 1). In 2006, the most frequent specific mood disorder diagnosis was "unspecified episodic mood disorder" (ICD-9-CM 296.90) and accounted for 11.0% of the hospitalizations for mood disorders (Table 2). This was followed by depressive disorder not elsewhere classified (311) and manic-depressive not otherwise specified (296.80) which accounted for 10.3 and 8.4 percent of the hospitalizations, respectively. The eight most frequent specific diagnoses accounted for over 50% of the hospitalizations with a principal diagnosis of mood disorders.

**Table 2:** The leading ICD-9-CM diagnoses in children hospitalized with a principal diagnosis of mood disorder as a percentage of all hospitalizations with a principal diagnosis of mood disorder, 2006

Diagnosis (ICD-9-CM code)^1^	CCS-MHSA Sub-category	Percentage of hospitalizations for mood disorders and 95% CI of estimate
Unspecified episodic mood disorder (296.90)	Bipolar	11.0 (10.8-11.3)

Depressive disorder not elsewhere classified (311)	Depressive	10.3 (10.1-10.5)

Manic-depressive not otherwise specified (296.80)	Bipolar	8.4 (8.2-8.6)

Depressive affective disorders - unspecified (296.2)	Depressive	6.6 (6.4-6.8)

Recurrent depressive disorder - severe (296.33)	Depressive	5.4 (5.3-5.7)

Depressive psychosis -severe (296.23)	Depressive	4.1 (4.0-4.4)

Recurrent depressive disorder - unspecified (296.30)	Depressive	2.4 (2.3-2.6)

Bipolar affective disorder, most recent episode mixed - unspecified (296.60)	Bipolar	2.3 (2.2-2.6)

^1 ^The categorization of ICD-9 codes into sub-categories, Bipolar and Depressive, is shown in Appendix 1.

The diagnosis of mood disorder was strongly associated with suicide attempt (or self-injurious behavior). Within children with any diagnosis of mood disorder, the percentage with a suicide attempt was 11.0% in 2000, 10.2% in 2003, and 9.7% in 2006. Within children with no diagnosis of mood disorder, the percentage with a suicide attempt was 0.2%, 0.1% and 0.1% in the same study years. In 2000, children with any diagnosis of mood disorder were 73 times more likely to have a code of "suicide attempt" on their hospital record compared to children without a diagnosis of mood disorders, in 2003 they were 101 times as likely and in 2006 they were 122 times as likely.

**Figure 1:**
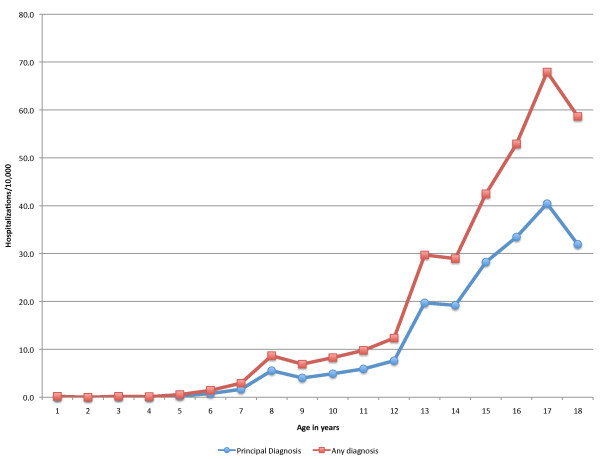
**Pediatric hospitalizations with diagnoses of mood disorders, age specific rates/10,000 children 2006**.

The incidence of hospitalizations for mood disorders increased with age. In 2006, the incidence of hospitalizations with any diagnosis of mood disorders was 7.2/10,000 in children ages 5-11 and 47.1/10,000 in children ages 12-17, and the incidence of hospitalizations with principal diagnosis of mood disorders was 4.4/10,000 and 29.0/10,000, respectively. The rate was less than 1.0/10,000 in children under 4. Age specific rates show a sharp increase between age 12 and 17, and a slight decline between age 17 and 18 (Figure 1). Among the hospitalizations with any diagnosis of mood disorder there were more females than males (57% female in 2006).

Over the years 2000 to 2006, an increasing proportion of hospital stays for mood disorders was paid by the government (Table 3). Medicare and Medicaid were expected payers for 35% of cases in 2000, increasing to 45% in 2006, and, correspondingly, the proportion paid by private insurance decreased from 57% to 45%. Over the same period, teaching hospitals accounted for an increasingly greater proportion of the hospitalizations, from 52 to 63%. The distribution of mood disorder hospitalizations by hospital size remained fairly constant (9-10% in small hospitals, 22-24% in medium hospitals, and 68% in large hospitals over 2000-2006). Inflation-adjusted charges for hospitalization increased from $10,600 in 2000, to $13,700 in 2003, to $16,300 in 2006, accompanied by a slight increase in length of stay from 7.1 days in 2000 to 7.7 days in 2006. The aggregate charges for hospitalizations with any diagnosis of mood disorders were over $2.2 billion in 2006.

**Table 3:** Characteristics of hospitalization among children under 18 with any mood disorder diagnosis, 2000-2006^1^

	2000	2003	2006
Primary expected payer			

Medicare or Medicaid	35.1%	40.2%	45.2%

Private	56.5%	49.7%	45.3%

Other	8.4%	9.8%	9.5%

Teaching status of hospital			

Teaching	51.7%	58.3%	62.8%

Non-teaching	48.4%	41.7%	37.2%

Hospital size			

Small	9.1%	10.1%	10.4%

Medium	24.0%	22.1%	21.6%

Large	66.9%	67.7%	68.0%

Average Length of Stay and 95% Confidence Intervals in days	7.0 (6.9-7.1)	7.1 (7.0-7.2)	7.6 (7.5-7.7)

Mean total charges ^2^	$10,578	$13,676	$16,287

^1 ^All differences were statistically significant at 0<0.001 except for the differences in length of stay

^2 ^Adjusted for inflation to 2006 dollars

**Figure 2:**
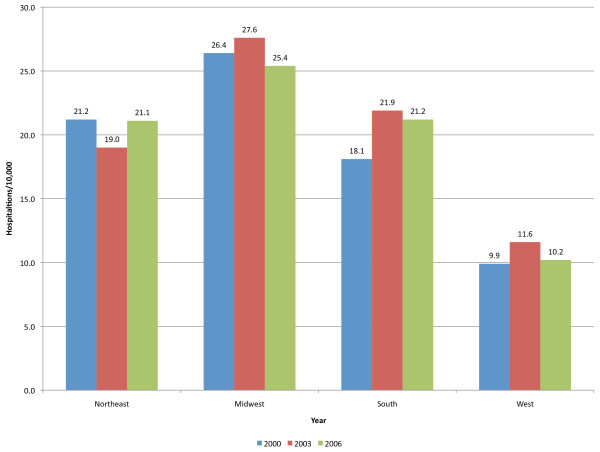
**Hospitalization rates with any diagnosis of mood disorders by region 2000-2006**.

Hospitalization rates for children with a principal diagnosis of mood disorders varied several fold by region of the country. The western region of the United States experienced the lowest pediatric hospitalization rates for mood disorders, ranging from 9.9/10,000 to 11.6/10,000 during the 2000-2006 time period (Figure 2). In the same period, hospitalization rates for mood disorders ranged from 18.1/10,000 to 21.9/10,000 in the South and 19.0/10,000 to 21.2/10,000 in the Northeast. Hospitalization rates for mood disorders in children were highest in the Midwest ranging from 25.4/10,000 to 27.6/10,000 children. Rates in the Midwest, Northeast and South were more than double the rates of the West. In the Midwest, the Relative Risk of admission to a hospital with a diagnosis of mood disorder was 2.7, 2.4 and 2.5 in the three study years. In the Northeast, these same Relative Risks were 2.1, 1.6 and 2.1, and in the South, the Relative Risks were 1.8, 1.9 and 2.1. In 2006, a similar pattern was observed for hospitalizations with any mental health diagnosis as a primary diagnosis with rates of 20.1/10,000 in the Midwest, 16.6/10,000 in the Northeast, and 16.5/10,000 in the South, all, higher than the 6.4/10,000 observed in the West. Hospitalizations with any mental health diagnosis (primary or not) were 49.7/10,000 in the Midwest, 51.6/10,000 in the Northeast, 48.5/10,000 in the South and 30.7/10,000 in the West. The regional variation in hospitalizations for mood disorders contrasts with the overall rates of pediatric hospitalizations by region for 2006. The highest hospitalization rates were found in the South (1,004.4/10,000) followed by the Northeast (891.4/10,000) and West (862.1/10,000), and lowest in the Midwest (788.1/10,000).

The mean age ranged from 13.9 in the South to 14.5 in the West. In 2006, the rates of hospitalizations for females and males followed the regional pattern; females and males from the Midwest had the highest rates and their counterparts from the West had the lowest rates of hospitalization with any diagnosis of mood disorder. The proportion paid by Medicare or Medicaid ranged from 31.2% in the West to 51.8% in the South, and the proportion paid by private insurance ranged from 38.3% in the South to 56.5% in the West. Mean total charges in 2006 were lowest in the Midwest ($12,260) and highest in the West ($23,980). The average length of stay was lowest in the Midwest (6.5 days) and highest in the Northeast (10.4 days).

## Discussion

The population rate of pediatric acute hospitalizations with a principal discharge diagnosis of mood disorder remained relatively stable from 2000 (12.4/10,000) through 2006 (12.1/10,000), even though the total number of hospitalizations increased in concert with the increase in the U.S. population. Although the 95% confidence intervals for the 2000, 2003 and 2006 estimates are extremely narrow and the difference in rates are statistically significant at the level of alpha = 0.05, the differences in rates are small and may not be significant from a clinical or public health perspective.

When the broad category of mood disorders is broken into the sub-categories of bipolar and depressive disorders two different patterns emerge. There was an increase in the rate of hospitalization with a principal diagnosis of bipolar disorders from 3.3/10,000 in 2000 to 5.5/10,000 children in 2006, and a concomitant decrease in hospitalizations with a principal diagnosis of depressive disorder from 8.9/10,000 to 6.2/10,000 from 2000 to 2006. In this database, the use of bipolar disorder diagnoses may be replacing the use of depressive disorder diagnoses, resulting in a relatively constant incidence of mood disorders hospitalizations over the time period, but further study may be required to explain these trends. A study of a similar data set in an earlier time period found admissions for both bipolar and depressive disorders to increase as a proportion of mental health admissions to community hospitals from 1990-2000, but did not calculate hospitalization rates relative to the denominator of children in the population [11]. Another study of hospital discharges in the US reported increases in both diagnoses as a proportion of psychiatric hospitalizations, and reported population based rates for the bipolar diagnoses only [20]. A study of outpatient office visits showed an increase in diagnosis and treatment of bipolar disorders between1994-1995 and 2002-2003, but did not report on depressive disorders [2]. In contrast to our findings, researchers studying hospitalizations in Germany between 2000 and 2007 found increase in population-based admission rates for both bipolar and depressive disorders [21].

When considering specific ICD-9-CM diagnoses, the three most frequently used diagnoses were "other and unspecified episodic mood disorder", "depressive disorder not elsewhere classified" and "manic-depressive not otherwise specified". Mood disorder hospitalizations were strongly linked to "suicide attempts", although at least a fourth of hospitalizations for suicide attempts and self-injurious behavior did not have a discharge diagnosis of mood disorder. This can be explained by the fact that suicidal behavior can occur in contexts other than mood disorder, such as personality disorders, substance abuse, or adjustment disorders [3]. The proportion of mood disorder hospitalizations paid by the government as well as the increasing trend between 2000 and 2006 is similar to that observed for all pediatric hospitalizations in this data set, 37%, 41% and 44%, respectively.

We found substantial regional variation in the rate of pediatric hospitalizations with a mood disorder diagnosis; in 2006, the rate was 2.5 times higher in the Midwest, 2.1 times higher in the Northeast and 1.8 times higher in the South than in the West. The regional differences were observed for all study years, 2000, 2003, and 2006. This finding is consistent with previous research showing a high proportion of mental health hospitalizations in the Midwest and the lowest proportion occurring in the West [11]. Our data go beyond the earlier analysis by using the hospitalization data to calculate population based rates. Other aspects of mental health care utilization have been examined by region, but do not supply ready explanations for the difference in hospitalization rates that we observed. Geographic variation in ambulatory care use (physician, other provider and emergency department visits) has been reported in adults for mental health/substance abuse, average spending and percentage paid out of pocket, showing the highest use in the Northeast and Midwest [22]. No statistically significant regional differences in antidepressant use in children and adolescents have been reported [23,24]. In contrast, Doshi and colleagues (2005) found rates of emergency department visits for suicide attempt or self-inflicted injury to be lowest in the Midwest, and highest in the West and Northeast, but the 95% confidence intervals of the estimates were wide and overlapping [25]. Their population ranged in age from under 14 to over 50, with a mean age of 31, and they did not analyze the regional data by age sub-groups. Blanco et al. (2008) estimated the prevalence of psychiatric disorders in college age youth to range from 41% in the Northwest to 53% in the Midwest [26].

It is difficult to compare our regional data to those from previous studies, because of differences in definitions, populations, and measures, and to explain the regional differences we observed in hospitalization rates without further detailed analyses of the underlying distribution of mood and mental health disorders, practice patterns, bed availability (including distribution of psychiatric hospitals), insurance policies, and other organizational factors that may affect hospitalization rates. In the HCUP KID, other mental health diagnoses appear to be higher in the Midwest and lower in the West, but general pediatric hospitalizations do not follow this pattern.

The strengths of this analysis lie in the large database, the probability based sampling, and the standardized methodology of the tri-annual data. As with other administrative measures of disease, hospital discharge diagnoses are subject to misclassification, and may either under- or over-estimate a given condition. Misclassification might also apply to other variables, such as suicidal behavior. One of the limitations is the lack of information about specific hospital units such as psychiatric or pediatric acute care units. The observation of hospitalizations for poisonings categorized as "suicide attempts", but without the diagnosis of mood disorders deserves further analysis to ascertain that mood disorders were not present, resulting in an underestimate of the true rate. Furthermore, the HCUP KID database does not include hospitalizations in psychiatric hospitals, substance abuse facilities, and rehabilitation hospitals (both long term and short term) and our analyses thus underestimate population rates of hospitalization for mood disorders. It is also possible that trends in hospitalization rates to psychiatric hospitals for mood disorders show differing patterns than that observed in general hospitals, but it does not detract from a central point, that large amounts of resources in general hospitals are being used to address mood disorders in children under 18 in the United States. These data have internal validity for inferences made about mood disorder hospitalizations in the United States between 2000-2006, but may not allow inferences to hospitalizations in psychiatric hospitals in the United States, and may not be generalizable outside of the United States.

## Conclusions

Mood disorders are a major reason for hospitalization during development, especially in adolescence. The mood disorder hospitalization rate remained relatively constant from 2000-2006, but with a decrease in the rate of depressive disorders hospitalizations and an increase in the rate of bipolar disorders hospitalizations. These data underscore the prominent burden of mood disturbances on the health of children and especially adolescents, trends in cost and utilization, the increasing burden on public resources, and regional variation. While we were unable to explain the regional variation in utilization of mental health inpatient care, we demonstrated variation that persisted over the study years 2000-2006. The data point to the need, on one hand, to provide inpatient specialized care for pediatric mood disorders, and, on the other hand, to develop more effective interventions to prevent or treat these conditions in the community thus decreasing the need for hospitalization.

## Competing interests

The authors declare that they have no competing interests.

## Authors' contributions

All authors contributed to discussion and interpretation of data analysis, and writing and revisions of the manuscript. TL identified the research question, provided epidemiologic expertise, and led the analysis and manuscript preparation. AK conducted the SAS programming for the data analysis and provided expertise in clinical psychology. AE provided expertise on HCUP KID and data analysis of HCUP KID. BV provided expertise on psychiatry and mental health in children.

## Appendix 1

**The single level CCS-MHSA category, 657, Mood disorders, and the ICD-9 codes that comprise the multi-level categories, Bipolar disorders, and Depressive disorders**.

Bipolar

296.00 MANIC DISORDER-UNSPECIFIED

296.01 MANIC DISORDER-MILD

296.02 MANIC DISORDER-MODERATE

296.03 MANIC DISORDER-SEVERE

296.04 MANIC DISORDER-SEVERE WITH PSYCHOTIC BEHAVIOR

296.05 MANIC DISORDER - PARTIAL REMISSION

296.06 MANIC DISORDER - FULL REMISSION

296.10 RECURRENT MANIC DISORDER-UNSPECIFIED

296.11 RECURRENT MANIC DISORDER-MILD

296.12 RECURRENT MANIC DISORDER-MODERATE

296.13 RECURRENT MANIC DISORDER-SEVERE

296.14 RECURRENT MANIC DISORDER-SEVERE WITH PSYCHOTIC BEHAVIOR

296.15 RECURRENT MANIC DISORDER-PARTIAL REMISSION

296.16  RECURRENT MANIC DISORDER-FULL REMISSION

296.40  BIPOLAR AFFECTIVE DISORDER MANIC-UNSPECIFIED

296.41  BIPOLAR AFFECTIVE DISORDER MANIC-MILD

296.42  BIPOLAR AFFECTIVE DISORDER MANIC-MODERATE

296.43  BIPOLAR AFFECTIVE DISORDER MANIC-SEVERE

296.44  BIPOLAR MANIC-SEVERE WITH PSYCHOTIC BEHAVIOR

296.45  BIPOLAR AFFECTIVE DISORDER MANIC-PART REMISSION

296.46  BIPOLAR AFFECTIVE DISORDER MANIC-FULL REMISSION

296.50  BIPOLAR AFFECTIVE DISORDER DEPRESSED-UNSPECIFIED

296.51  BIPOLAR AFFECTIVE DISORDER DEPRESSED-MILD

296.52  BIPOLAR AFFECTIVE DISORDER DEPRESSED-MODERATE

296.53  BIPOLAR AFFECTIVE DISORDER DEPRESSED-SEVERE

296.54  BIPOLAR DEPRESSED-SEVERE WITH PSYCHOTIC BEHAVIOR

296.55  BIPOLAR AFFECTIVE DEPRESSED-PARTIAL REMISSION

296.56  BIPOLAR AFFECTIVE DEPRESSED-FULL REMISSION

296.60  BIPOLAR AFFECTIVE DISORDER MIXED-UNSPECIFIED

296.61  BIPOLAR AFFECTIVE DISORDER MIXED-MILD

296.62  BIPOLAR AFFECTIVE DISORDER MIXED-MODERATE

296.63  BIPOLAR AFFECTIVE DISORDER MIXED-SEVERE

296.64  BIPOLAR MIXED-SEVERE With PSYCHOTIC BEHAVIOR

296.65  BIPOLAR AFFECTIVE DISORDER MIX-PARTIAL REMISSION

296.66  BIPOLAR AFFECTIVE DISORDER MIX-FULL REMISSION

296.7  BIPOLAR AFFECTIVE NOT OTHERWISE SPECIFIED

296.80  MANIC-DEPRESSIVE NOT OTHERWISE SPECIFIED

296.81  ATYPICAL MANIC DISORDER

296.82  ATYPICAL DEPRESSIVE DISORDER

296.89  MANIC-DEPRESSIVE NOT ELSEWHERE CLASSIFIED

296.90  UNSPECIFIED EPISODIC MOOD DISORDER

296.99  AFFECTIVE PSYCHOSES NOT ELSEWHERE CLASSIFIED

Depressive 

293.83 ORGANIC AFFECTIVE SYNDROME

296.20  DEPRESSIVE AFFECTIVE DISORDERS-UNSPECIFIED

296.21  DEPRESSIVE AFFECTIVE DISORDER-MILD

296.22  DEPRESSIVE AFFECTIVE DISORDER-MODERATE

296.23  DEPRESSIVE AFFECTIVE DISORDER-SEVERE WITHOUT PSYCHOTIC BEHAVIOR

296.24  DEPRESSIVE AFFECTIVE DISORDER-SEVERE WITH PSYCHOTIC BEHAVIOR

296.25  DEPRESSIVE AFFECTIVE DISORDER-PARTIAL REMISSION

296.26  DEPRESSIVE AFFECTIVE DISORDER-FULL REMISSION

296.30  RECURRENT DEPRESSIVE DISORDER-UNSPECIFIED

296.31  RECURRENT DEPRESSIVE DISORDER-MILD

296.32  RECURRENT DEPRESSIVE DISORDER-MODERATE

296.33  RECURRENT DEPRESSIVE DISORDER-SEVERE

296.34  RECURRENT DEPRESSIVE DISORDER-SEVERE WITH PSYCHOTIC BEHAVIOR

296.35  RECURRENT DEPRESSIVE DISORDER-PARTIAL REMISSION

296.36  RECURRENT DEPRESSIVE DISORDER-FULL REMISSION

   3004  NEUROTIC DEPRESSION

     311  DEPRESSIVE DISORDER NOT ELSEWHERE CLASSIFIED
